# The factors affecting breastfeeding self-efficacy, LATCH scores and effect of postnatal breastfeeding education on mothers’ self-efficacy*

**DOI:** 10.1590/1980-220X-REEUSP-2024-0281en

**Published:** 2025-05-02

**Authors:** Aslı Karakuş Selçuk, Emre Yanikkerem, Seda Ünalmiş Erdoğan

**Affiliations:** 1 Manisa Celal Bayar University, Faculty of Health Science, Nursing Department, Manisa, Turkey.; 2 Manisa Celal Bayar University, Hafsa Sultan Hospital, Manisa, Turkey.

**Keywords:** Breast Feeding, Self Efficacy, Education, Postpartum Period, Aleitamento Materno, Autoeficácia, Educação, Período Pós-Parto

## Abstract

**Objective:**

To identify the factors affecting breastfeeding self-efficacy and LATCH scores and to assess the impact of postnatal breastfeeding education on mothers’ self-efficacy.

**Method:**

This study employed a quasi-experimental pretest-posttest design with a single group. It was completed with 217 mothers who gave birth in a hospital in Turquía between May 2023 and December 2023. Data were collected using Breastfeeding Self-efficacy Scale, and LATCH assessment tool. Breastfeeding education was given to mothers before discharge. Breastfeeding self-efficacy was re-evaluated on the phone four weeks after discharge.

**Results:**

The mean LATCH score among mothers was 9,2 ± 1.3 (min = 3, max = 10). Higher LATCH scores were observed in mothers with primary or secondary education, those who were unemployed, had four or more pregnancies and children, breastfed within the first hour, and had prior breastfeeding experience before discharge. Mothers with breastfeeding experience and those with four pregnancies and three children had higher breastfeeding self-efficacy scores before discharge. Four weeks after discharge was given to mothers, the mean breastfeeding self-efficacy score increased significantly from 58,9 ± 6,7 to 68,4 ± 1,9. A statistically significant improvement in breastfeeding self-efficacy was observed only among mothers with prior breastfeeding experience four weeks after discharge.

**Conclusion:**

Regular breastfeeding education in the early postpartum period is crucial, particularly for young, primiparous mothers with higher education levels, those who are employed, with a history of cesarean deliveries, or lack of prior breastfeeding experience. The findings indi-cate that breastfeeding education positively impacts mothers’ breastfeeding self-efficacy.

## RESUMO

**Objetivo:**

Identificar os fatores que afetam a autoeficácia na amamentação e as pontuações LATCH e avaliar o impacto da educação pós-natal sobre a amamentação na autoeficácia das mães.

**Método:**

Este estudo empregou um delineamento quase experimental de pré-teste-pós-teste com um único grupo. Foi concluído com 217 mães que deram à luz em um hospital em Turquía entre maio de 2023 e dezembro de 2023. Os dados foram coletados usando a Breastfeeding Self-efficacy Scale e a ferramenta de avaliação LATCH. A educação sobre amamentação foi dada às mães antes da alta. A autoeficácia na amamentação foi reavaliada por telefone quatro semanas após a alta.

**Resultados:**

A pontuação média do LATCH entre as mães foi de 9,2 ± 1,3 (mín. = 3, máx. = 10). Pontuações LATCH mais altas foram observadas em mães com educação primária ou secundária, aquelas que estavam desempregadas, tiveram quatro ou mais gestações e filhos, amamentaram na primeira hora e tiveram experiência anterior com amamentação antes da alta. Mães com experiência em amamentação e aquelas com quatro gestações e três filhos apresentaram maiores escores de autoeficácia em amamentação antes da alta. Quatro semanas após a alta, a pontuação média de autoeficácia em amamentação aumentou significativamente de 58,9 ± 6,7 para 68,4 ± 1,9. Uma melhora estatisticamente significativa na autoeficácia em amamentação foi observada apenas entre mães com experiência anterior em amamentação quatro semanas após a alta.

**Conclusão:**

A educação regular sobre amamentação no período pós-parto inicial é crucial, principalmente para mães jovens, primíparas, com níveis de educação mais altos, aquelas que estão empregadas, com histórico de partos cesáreos ou falta de experiência anterior em amamentação. Os resultados indicam que a educação sobre amamentação impacta positivamente a autoeficácia em amamentação das mães.

## INTRODUCTION

It is universally recognized that breast milk is the optimal source of nutrition for newborns. The United Nations Children’s Fund (UNICEF) recommends that mothers initiate breastfeeding within the first hour after birth, exclusively breastfeed for the first six months, and continue breastfeeding until the child reaches at least two years of age or beyond^(1)^. Despite these well-documented advantages, global data indicate that only 48% of infants aged 0–5 months are exclusively breastfed. This rate varies significantly by region, reaching 60% or higher in South Asia but dropping to 26% in North America^(2)^. According to the latest data from the Türkiye Population Health Survey (2018), although the majority of children under two years of age (71.0%) were breastfed within one hour of birth, 41.0% of babies under 6 months were exclusively breastfed^(3)^.

Self-efficacy refers to an individual’s perception of their ability to perform a specific action or behavior. In the context of breastfeeding, this perception is defined as breastfeeding self-efficacy (BSE)^(4)^. The mother’s self-efficacy can affect the woman’s initiation and continuation of breastfeeding, how she will cope with difficulties in this process, and the mother’s breastfeeding decision^(5)^. National and international studies have highlighted several key factors that impact breastfeeding self-efficacy in the postpartum period. These include the number of births^(6)^, prior breastfeeding experience^(7)^, exclusive breastfeeding status^(7−10)^, receiving breastfeeding counseling before or after delivery^(7)^, number of children^(11)^, mode of delivery (spontaneous vaginal birth)^(12)^, breastfeeding difficulties^(5)^, the newborn’s feeding pattern in the previous 24 hours (exclusive, partial, or no breastfeeding)^(5)^, perceived insufficiency of milk supply^(5)^, and the primary caregiver’s attitude toward breastfeeding^(5)^.

The literature indicates that breastfeeding educational programs and supportive interventions provided to mothers during the antenatal^(13,14)^ and postpartum periods^(15-21)^ are effective in improving LATCH scores and BSE. Studies conducted in Türkiye have shown that breastfeeding education delivered using the teach-back method^(17)^ and tele-education on breastfeeding^(16)^ enhanced mothers’ breastfeeding success and BSE. Similarly, research in Japan^(21)^ found that a breastfeeding self-care program increased both BSE and breastfeeding continuation, while studies in China^(20)^ demonstrated that improving self-efficacy significantly impacted the rate of exclusive breastfeeding.

The key aspect that differentiates this study from previous research is its evaluation of both the effectiveness of postnatal breastfeeding education on BSE and the factors influencing BSE, combined with a large sample size. The objectives of this study were: to identify the factors affecting breastfeeding self-efficacy and LATCH scores and to assess the impact of postnatal breastfeeding education on mothers’ self-efficacy.

### Research Questions

- How do mothers’ characteristics influence BSE and LATCH scores before discharge?
- How do mothers’ characteristics influence BSE four weeks after discharge?
- Is there a difference in pre-and post-test education measures on BSE scores?

## METHOD

### Design of Study

This study employed a quasi-experimental pretest-posttest design with a single group.

### Sample Size Calculation

The universe of the study consisted of 882 mothers who gave birth in the obstetrics and gynecology clinic of a university hospital in Türkiye between May 1, 2023, and December 31, 2023. The universally known population formula was used to determine the minimum sample size. Using this formula, the minimum sample size was calculated as 213 women with the Epi Info 2000 statistical software (90% confidence interval, 50% unknown prevalence, and 5% margin of error). The sample size was further calculated as 207 (a medium effect size of 0.3, a 5% alpha error probability, and 99% power) using the G*Power program. The study was completed with 217 mothers, and a post-hoc power analysis was performed again. The effect size was 1.427, and the power was 1.00^(22)^.

### Inclusion and Exclusion Criteria

The study included mothers who were over 18 years of age, were breastfeeding, and those who had healthy infants. Mothers with visual or hearing impairments, psychiatric illnesses, multiple births, infants weighing less than 2500 g or born prematurely, those who did not speak Turkish, and those who did not voluntarily participate were excluded from the study.

### Data Collection Tools

The questionnaire consisted of three sections. The first section included 19 questions covering the mothers’ sociodemographic and obstetric characteristics, such as age, education level, income, employment status, mode of delivery, number of births, time of breastfeeding initiation, newborn’s weight, and gender. This questionnaire was developed based on the existing literature^(6-11)^. The second section featured the LATCH Breastfeeding Assessment Tool, a scale developed by Jensen, Wallace, and Kelsay^(23)^ to assess mothers’ breastfeeding behaviors^(23)^. Each item on the scale consists of five criteria, evaluated with scores of 0, 1, or 2 points. LATCH is an acronym representing the key components of breastfeeding diagnosis and assessment:

- L (Latch on breast): How well the newborn attaches to the breast
- A (Audible Swallowing): Detecting the newborn’s swallowing sounds
- T (Type of Nipple): Nipple type
- C (Comfort of Breast/Nipple): The mother’s breast/nipple comfort
- H (Hold/Position): The level of assistance required for the mother to position her newborn for sucking^(23,24)^.

The LATCH scale ranges from 0 to 10, with higher scores indicating greater breastfeeding success. Scoring is determined by observing the mother’s breastfeeding behavior. A Turkish validity and reliability study of this tool was conducted by Yenal and Okumuş (2003), with a reported Cronbach’s alpha value of 0.95, indicating high reliability. The third section included the Breastfeeding Self-Efficacy Scale-Short Form (BSES-SF). Originally developed by Dennis and Faux^(25)^ with 33 items, the scale was revised in 2003 into a 14-item short form^(26)^. This five-point Likert-type scale ranges from 1 (not at all confident) to 5 (very confident), with total scores ranging from 14 to 70. Higher scores indicate greater BSE. The Turkish validity and reliability study of the scale was conducted by Tokat et al.^(4)^, with a Cronbach’s alpha value of 0.86, demonstrating good reliability.

### Data Collection

The data was collected in three sequential stages, each of which had an assigned researcher. In the first stage, data were collected by face-to-face interviews in the mothers’ rooms. During this 10-minute phase, mothers’ mobile phone numbers were recorded, and data were collected using the sociodemographic and obstetric characteristics questionnaire and the BSES-SF forms. They were informed that they would be contacted four weeks later. In the second phase, women’s breastfeeding success was assessed using the LATCH scale, and they received breastfeeding education. In the final phase, mothers were contacted by phone four weeks after discharge and the mothers’ BSE was re-evaluated with BSES-SF.

### Intervention

At the hospital where the study was conducted, the minimum hospital stay is 24 hours for mothers who give birth vaginally and 48 hours for those who undergo a cesarean section. In this baby-friendly hospital, mothers begin breastfeeding their newborns within 30 minutes after birth, all mothers receive breastfeeding education, and support is available throughout their hospital stay. The same structured breastfeeding education program, developed based on the literature^(1,2,20,27)^, was followed for each mother. The third researcher delivered a 20-minute lecture, which included information on breast structure, the lactation mechanism, the latch-on position, milk production, soothing techniques for infants, the importance and composition of breast milk, the benefits of breastfeeding for both mother and infant, breastfeeding frequency and duration, common breastfeeding problems and suggested solutions, the nutritional needs of breastfeeding mothers, and the expression and storage of breast milk. The topics addressed were based on the most common questions raised by mothers during the early postpartum period. Through the education, mothers’ attention was directed to their knowledge and skills that facilitate successful breastfeeding (verbal persuasion) and how they could achieve successful breastfeeding experiences (performance accomplishments) by applying these skills. Breastfeeding techniques were demonstrated practically, and mothers were encouraged to ask questions. Finally, the researcher provided positive feedback (verbal persuasion) using their professional knowledge and expertise. The third researcher, a certified breastfeeding nurse working in this clinic, regularly receives training on breastfeeding. She visited all mothers’ rooms during their hospital stay, providing one-on-one breastfeeding education on the first postpartum day.

### Data Analysis and Treatment

The statistical analysis was performed using the SPSS 20.0 program. Frequency distributions were used to assess the descriptive characteristics. The normality distribution of the data was determined by Skewness and Kurtosis tests. Since the data was not normally distributed, the relationship between the independent variables of the study and LATCH, and BSE scores were evaluated with Kruskal Wallis, Mann-Whitney U, and Tamhane’s T2 test. The Wilcoxon test was used to determine the difference in pre and post-test measures on BSE scores. A p-level of < 0.05 was considered statistically significant.

### Ethical Aspects

Ethical approval was obtained from the Ethical Committee of Manisa Celal Bayar University (Date: 12.04.2023, Number: 20.478.486/1803). Permission was taken from the institution to collect the data for the study. Written and verbal informed consent was obtained from all mothers. Permission was received from Yenal for the BSES-SF and from Tokat for the LATCH Turkish version to use in this study.

## RESULTS

**Figure 1:**
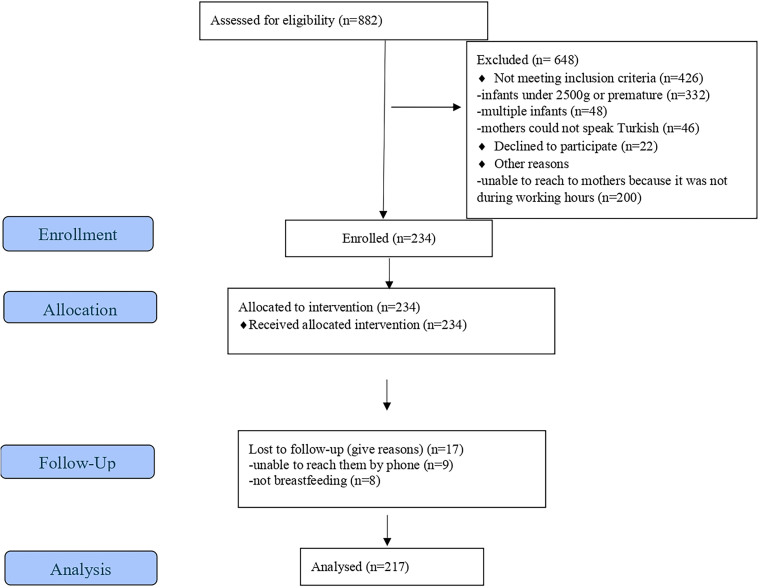
Flow diagram of study participants.

In the study, 648 out of 882 mothers who gave birth in the hospital during the data collection period were excluded. In the first stage, data were collected from 234 mothers, and in the second stage, breastfeeding education was provided. In the third stage, mothers who did not respond to phone calls (n = 9) and those who did not breastfeed (n = 8) were excluded from the study. The study was completed with 217 mothers. A diagram showing the participant flow was presented in Figure 1.

### Descriptive Characteristics of Mothers

In the study, the mean age of the mothers was 28.8 ± 5.5 (min = 18, max = 43), and about half of the mothers (47.0%) were between 26 and 33 years old. Overall, 43.8% had a primary or secondary school education, 79.3% were unemployed, and 68.2% reported that their family income was equal to their expenses. Most of the mothers (86.2%) had planned pregnancies, 88.0% had a cesarean birth, about half of the mothers were experiencing their first pregnancy (41.9%), were primiparous (47.5%), and 59.4% had diseases during pregnancy. The mean birth weight of the infants was 3175.9 ± 480.9 (min = 2500, max = 4600). Of the mothers, 86.2% breastfed their infants within the first hour. Most mothers (81.1%) stated that they had not received breastfeeding training, and 46.1% had prior breastfeeding experience (Table 1).

**Table 1:** – Descriptive characteristics of mothers- Manisa, Türkiye, 2023.

Characteristics of mothers	n	%
**Age groups of mothers**		
18–25 years	66	30.4
26–33 years	102	47.0
34–43 years	49	22.6
**Education status of mothers**		
Literate/Not illiterate	9	4.1
Primary/Secondary school	95	43.8
High school	61	28.1
University and above	52	24.0
**Employment status of mothers**		
Employed	45	20.7
Unemployed	172	79.3
**Perceived income status**		
Income is lower than expenses	54	24.9
Income is equal to expenses	148	68.2
Income is higher than expenses	15	6.9
**The number of pregnancies**		
1	91	41.9
2	41	18.9
3	40	18.4
4 or more	45	20.7
**The number of children**		
1	103	47.5
2	44	20.3
3	41	18.9
4 or more	29	13.4
**The type of birth**		
Cesarean birth	191	88.0
Vaginal birth	26	12.0
**Newborn’s gender**		
Female	102	47.0
Male	115	53.0
**Newborn’s birth weight**		
2500–3000	82	37.8
3001–3500	93	42.9
3501 or more	42	19.4
**Current pregnancy**		
Planned	187	86.2
Unplanned	30	13.8
**Had disease during pregnancy**		
Yes	88	40.6
No	129	59.4
**Received any breastfeeding education**		
Yes	41	18.9
No	176	81.1
**Breastfeeding experiences of mothers**		
Yes	100	46.1
No	117	53.9
**Time of breastfeeding initiation**		
First 1 hour	187	86.2
More than 1 hour	30	13.8
**Total**	**217**	**100.0**

### The Relationship Between Mothers’ Descriptive Characteristics and Latch Scores Before Discharge

In this study, the mean LATCH score of the mothers was 9.2 ± 1.3 (min = 3, max = 10). The mean LATCH score was significantly higher in mothers with primary or secondary education and those who were literate or illiterate compared to those with university-level education or higher (p = 0.002). Unemployed mothers had higher LATCH scores than employed mothers (p = 0.001). The LATCH score was significantly higher in mothers who had three or four or more pregnancies compared to those with one or two pregnancies (p = 0.000). Similarly, the mean LATCH score was higher in mothers who had three children than in those with one or two children (p = 0.000). Mothers who breastfed their infants within the first hour (p = 0.044) and those who had previous breastfeeding experience (p = 0.000) had higher LATCH scores (Table 2).

**Table 2:** The relationship between mothers’ descriptive characteristics and LATCH scores before discharge – Manisa, Türkiye, 2023.

Characteristics of mothers (n: 217)	LATCH scores before discharge				
	Mean	SD	Median	IQR	Test/p
**Age groups of mothers**					
18–25 years	9.02	1.26	9.00	1.00	KW = 3.444
26–33 years	9.19	1.27	10.00	1.00	p = 0.179
34–43 years	9.31	1.21	10.00	1.00	
**Education status of mothers**					
Literate/Not illiterate (a)	9.67	0.50	10.00	1.00	KW = 14.921
Primary/Secondary school (b)	9.43	1.02	10.00	1.00	**p = 0.002**
High school (c)	9.13	1.19	10.00	1.00	*a > d, b > d
University and above (d)	8.63	1.60	9.00	2.00	
**Perceived income status**					
Income is lower than expenses	9.31	1.11	10.00	1.00	KW = 1.778
Income is equal to expenses	9.14	1.30	10.00	1.00	p = 0.411
Income is higher than expenses	8.93	1.22	9.00	2.00	
**Employment status of mothers**					
Employed	8.60	1.49	9.00	2.00	MU = 2693.000
Unemployed	9.31	1.14	10.00	1.00	**p = 0.001**
**Current pregnancy**					
Planned	9.12	1.29	10.00	1.00	MU = 2357.000
Unplanned	9.47	0.97	10.00	1.00	p = 0.121
**The type of birth**					
Cesarean birth	9.15	1.26	10.00	1.00	MU = 2275.500
Vaginal birth	9.30	1.19	10.00	1.00	p = 0.445
**The number of pregnancies**					
1 (a)	8.66	1.49	9.00	2.00	KW = 43.512
2 (b)	9.12	1.08	9.00	1.00	**p = 0.000**
3 (c)	9.75	0.59	10.00	0.00	*c > a, c > b, d > a, d > b
4 or more (d)	9.71	0.79	10.00	0.00	
**The number of children**					
1 (a)	8.72	1.47	9.00	2.00	KW = 36.243
2 (b)	9.27	1.02	10.00	1.00	**p = 0.000**
3 (c)	9.78	0.57	10.00	0.00	*c > a, c > b, d > a
4 or more (d)	9.69	0.81	10.00	0.00	
**Had disease during pregnancy**					
Yes	9.05	1.28	9.50	1.00	MU = 5114.500
No	9.25	1.24	10.00	1.00	p = 0.172
**Newborn’s gender**					
Female	9.18	1.30	10.00	1.00	MU = 5703.500
Male	9.16	1.22	10.00	1.00	p = 0.699
**Newborn’s birth weight**					
2500–3000	9.09	1.28	9.00	1.00	KW = 1.872
3001–3500	9.17	1.32	10.00	1.00	p = 0.392
3501 or more	9.31	1.07	10.00	1.00	
**Time of breastfeeding initiation**					
First 1 hour	9.23	1.20	10.00	1.00	MU = 2222.000
More than 1 hour	8.77	1.50	9.00	1.25	**p = 0.044**
**Received any breastfeeding education**					
Yes	90.2	1.37	9.00	1.00	MU = 3285.000
No	9.19	1.23	10.00	1.00	p = 0.324
**Breastfeeding experiences of mothers**					
Yes	9.63	0.73	10.00	1.00	MU = 3570.000
No	8.77	1.46	9.00	2.00	**p = 0.000**

IQR: Interquartile range, KW: Kruskal Wallis (test for independent samples), MU: Mann-Whitney U (two indenpendent sample test)

*Tamhane’s T2, SD: Standard deviation.

### The Relationship Between Mothers’ Descriptive Characteristics and Bses-Sf Scores Before and Four Weeks After Discharge

Mothers who had no diseases during pregnancy (p = 0.009), and those who had previous breastfeeding experience (p = 0.005), four pregnancies (p = 0.011), and three children (p = 0.007) were found to have higher BSES-SF scores before discharge. There were no significant differences between BSES-SF scores and age groups, education level, perceived income, employment status, type of birth, newborn gender, birth weight, or time of breastfeeding initiation before discharge. A significant difference in BSES-SF scores was found only in mothers with previous breastfeeding experience (p = 0.003) four weeks after discharge (Table 3).

**Table 3:** The relationship between mothers’ descriptive characteristics and BSES-SF scores before and four weeks after discharge – Manisa, Türkiye, 2023.

Characteristics of mothers (n: 217)	BSES-SF score									
	Before discharge					Four weeks after discharge				
	Mean	SD	Median	IQR	Test/p	Mean	SD	Median	IQR	Test/p
**Age groups of mothers**										
18–25 years	58.08	6.38	56.00	10.25	KW = 1.658	68.36	1.76	68.00	2.00	KW = 0.757
26–33 years	59.06	6.82	56.00	11.25	p = 0.436	68.54	1.79	68.00	2.00	p = 0.685
34–43 years	59.63	6.99	57.00	11.50		68.08	2.32	68.00	3.00	
**Education status of mothers**										
Literate/Not illiterate	60.44	7.23	58.00	14.00	KW = 3.003	67.67	4.12	68.00	2.00	KW = 3.358
Primary/Secondary	59.22	6.85	56.00	13.00	p = 0.391	68.64	1.62	70.00	2.00	P = 0.340
High school	59.09	6.60	56.00	11.00		68.28	2.02	68.00	2.50	
University and above	57.77	6.57	56.00	10.75		68.15	1.71	68.00	2.00	
**Perceived income status**										
Income is lower than expenses	59.39	7.01	59.00	12.25	KW = 0.452	68.06	2.32	68.00	3.00	KW = 2.549
Income is equal to expenses	58.82	6.58	56.00	11.00	p = 0.798	68.44	1.79	68.00	2.00	p = 0.280
Income is higher than expenses	57.73	7.43	56.00	13.00		69.00	1.25	70.00	2.00	
**Employment status of mothers**										
Employed	57.27	6.33	56.00	10.00	MU = 3220.000	68.09	2.03	68.00	2.00	MU = 3497.500
Unemployed	59.31	6.78	56.00	11.75	p = 0.082	68.46	1.88	68.00	2.00	p = 0.289
**Current pregnancy**										
Planned	58.66	6.59	56.00	11.00	MU = 2415.000	68.37	1.86	68.00	2.00	MU = 2593.500
Unplanned	60.30	7.46	57.50	13.75	p = 0.221	68.43	2.27	70.00	2.25	p = 0.480
**The type of birth**										
Cesarean birth	58.62	6.71	56.00	11.00	MU = 2037.000	68.36	1.95	68.00	2.00	MU = 2417.000
Vaginal birth	60.85	6.58	62.00	13.25	p = 0.136	68.54	1.68	69.00	3.00	p = 0.815
**The number of pregnancies**										
1 (a)	57.49	6.30	56.00	10.00	KW = 11.046	68.25	1.65	68.00	2.00	KW = 5.407
2 (b)	58.61	7.09	56.00	13.00	**p = 0.011**	68.15	2.38	68.00	2.00	p = 0.144
3 (c)	60.20	6.91	58.00	12.00	*d > a	68.70	2.11	70.00	2.00	
4 or more (d)	60.80	6.58	59.00	13.00		68.58	1.75	70.00	2.00	
**The number of children**										
1 (a)	57.75	6.37	56.00	11.00	KW = 12.182	68.16	2.02	68.00	2.00	KW = 3.632
2 (b)	58.27	6.76	56.00	12.50	**p = 0.007**	68.70	1.44	68.00	2.00	p = 0.304
3 (c)	61.31	6.95	61.00	14.00	*c > a	68.46	2.15	70.00	2.50	
4 or more (d)	60.45	6.71	57.00	13.00		68.59	1.78	70.00	2.00	
**Had disease during pregnancy**										
Yes	57.49	6.74	55.50	10.75	MU = 4499.000	68.24	1.75	68.00	2.00	MU = 5029.000
No	59.85	6.57	57.00	12.50	**p = 0.009**	68.48	2.01	69.00	2.00	p = 0.128
**Newborn’s gender**										
Female	58.82	6.46	56.00	11.00	MU = 5740.000	68.56	1.85	68.00	2.00	MU = 5324.500
Male	58.95	6.97	56.00	12.00	p = 0.786	68.23	1.96	68.00	2.00	p = 0.212
**Newborn’s birth weight**										
2500–3000	59.49	6.95	57.00	12.00	KW = 1.436	68.33	2.18	68.00	2.00	KW = 0.056
3001–3500	58.43	6.49	56.00	11.50	p = 0.488	68.43	1.75	68.00	2.00	p = 0.973
3501 or more	58.74	6.84	56.00	11.25		68.38	1.74	68.00	2.00	
**Time of breastfeeding initiation**										
First 1 hour	58.89	6.78	56.00	11.00	MU = 2762.500	68.41	1.87	68.00	2.00	MU = 2663.500
More than 1 hour	58.87	6.44	56.00	11.00	p = 0.894	68.17	2.21	68.00	2.25	p = 0.636
**Received any breastfeeding education**										
Yes	59.05	6.93	57.00	12.00	MU = 3606.500	68.34	2.42	69.00	2.00	MU = 3439.000
No	58.85	6.69	56.00	11.00	p = 0.997	68.39	1.78	68.00	2.00	p = 0.618
**Breastfeeding experiences of mothers**										
Yes	60.17	6.95	56.50	12.75	MU = 4545.000	68.74	1.74	70.00	2.00	MU = 4585.000
No	57.79	6.35	56.00	11.00	**p = 0.005**	68.08	2.00	68.00	2.00	**p = 0.003**

IQR: Interquartile range, KW: Kruskal Wallis (test for independent samples), MU: Mann-Whitney U (two indenpendent sample test)

*Tamhane’s T2, SD: Standard deviation.

### Bses-Sf Scores of Mothers Before and Four Weeks After Discharge

In this study, while the BSES-SF score of mothers was 58.9 ± 6.7 (min = 45, max = 70) before breastfeeding training, it increased statistically significantly to 68.4 ± 1.9 (min = 57, max = 70) four weeks after discharge (Data not shown). There was a significant difference between four weeks after discharge and before discharge in mothers’ BSE scores after the Wilcoxon Test was carried out (Z = −11.936 p = 0.000) and the results of the mothers’ BSE also indicate a positive gain (Table 4).

**Table 4:** BSES-SF scores of mothers before and four weeks after discharge – Manisa, Türkiye, 2023.

BSE four weeks after discharge-BSE before discharge (n: 217)	n	Mean rank	Sum of ranks	
Negative ranks	10	19.50	195.00	
Positive ranks	187	103.25	19308.00	Z = -11.936
Ties	20			* **p = 0.000**

*Wilcoxon test.

## DISCUSSION

The present study aimed to identify the factors influencing BSE and LATCH scores and to examine the impact of postnatal breastfeeding education on mothers’ self-efficacy. In this study, the mean LATCH score was 9.2 ± 1.3 (min = 3, max = 10). Considering that the LATCH scale ranges from 0 to 10, the results indicate that the average LATCH scores of mothers were high. A review of studies conducted in Türkiye found that the mean LATCH scores of mothers were 6.2 ± 1.6^(7)^, 7.3 ± 2.2^(12)^, 8.2 ± 1.8^(6)^, and 8.4 ± 1.5^(11)^. Similarly, studies from India and Italy reported mean LATCH scores of 7.2 ± 1.1^(28)^ and 7.3 ± 1.7^(29)^, respectively. Compared to these studies, the LATCH scores in the present study were higher, which can be attributed to the fact that the study was conducted in a baby-friendly hospital. It is believed that the breastfeeding education provided to mothers in the clinic contributed to this increase in LATCH scores. Additionally, the breastfeeding department nurse (the third researcher) working in this clinic regularly receives breastfeeding training and has extensive experience in providing practical breastfeeding education and support to mothers. Consistent with our findings, previous studies have shown that mothers with four or more pregnancies^(6)^, mothers with three children^(7,11)^, mothers with prior breastfeeding experience before discharge^(7)^, and mothers who exclusively breastfed before discharge^(28,29)^ had higher LATCH scores. These results highlight the crucial role of maternal experience in breastfeeding success. Prior breastfeeding experience enhances self-confidence, and familiarity with breastfeeding techniques leads to better breastfeeding outcomes. In the present study, those who initiated breastfeeding within the first hour had higher LATCH scores than those who started later. Similarly, Gercek et al.^(6)^ reported that those who started breastfeeding within 30 minutes of birth had higher LATCH scores than those who initiated it 1–4 hours or 5 or more hours after birth. This early initiation of breastfeeding may contribute to stronger maternal-infant bonding, a more supportive breastfeeding environment, and greater maternal motivation for successful breastfeeding. Additionally, mothers with primary or secondary education, those who were literate or not illiterate, and unemployed mothers had statistically significantly higher LATCH scores, due to the fact that there were more positive attitudes toward breastfeeding among women in these groups and the fact that unemployed mothers have more time and opportunities to breastfeed their infants. As observed in previous studies^(6,7,11)^, it is an expected outcome that mothers with previous pregnancies, children, and breastfeeding experience demonstrate greater breastfeeding success and higher LATCH scores.

In the present study, prior to the provided education, the mean BSES-SF score of the mothers was found 58.9 ± 6.7 (min = 45, max = 70). There were some studies in Türkiye in which the BSES-SF score varied between 54 and 57^(6,7,11,15,30)^. The mean BSES-SF score was 59.7 ± 6.3 in Brazil^(19)^, 48.2 ± 7.4 in China^(20)^, 47.3 ± 8.6 in Canada^(31)^, and 34.8 ± 1.1 in Japan^(21)^. The findings suggest that the BSES-SF score in this study is consistent with those of previous ones in Türkiye. However, the lower BSES-SF scores reported in studies from other countries may be attributed to cultural and sociodemographic differences. Consistent with our study, mothers with four or more pregnancies^(6)^ and those with three or more children^(7,11)^ had higher BSES-SF scores before discharge. Additionally, a significant increase in BSE scores was observed in mothers with prior breastfeeding experience four weeks after discharge. BSE is influenced by maternal experience and cultural background and tends to be higher in women with multiple pregnancies. Differences in breastfeeding rates and self-efficacy across countries may be linked to variations in social structures and healthcare support systems.

The findings of this study demonstrated a significant increase in the mean BSES-SF score four weeks after discharge (before discharge: 58.9 ± 6.7, four weeks after discharge: 68.4 ± 1.9). In the literature, studies were conducted with pretest-posttest randomized controlled quasi-experimental in different follow-up time intervals to evaluate mothers’ BSE. Previous randomized controlled and quasi-experimental studies with pretest-posttest designs conducted in Canada, China, Brazil, Japan, Egypt, and Türkiye have also found that breastfeeding education significantly improves BSE levels during the postpartum period^(16,17,19−21,31−34)^. Studies from Türkiye show that after breastfeeding education using the teach-back method, the mean BSES-SF score increased from 45.67 ± 11.65 before education to 67.02 ± 2.16^(17)^. Similarly, after tele-education on breastfeeding, the BSES-SF score increased from 43.54 ± 7.15 to 52.04 ± 6.25 four weeks later^(16)^. A quasi-experimental pretest-posttest study in Japan^(21)^ also found that a breastfeeding self-care program, which included a pamphlet and audiovisual materials, effectively increased BSE scores from 34.8 ± 1.1 before education to 49.9 ± 0.9 four weeks later. In China, a postnatal telephone follow-up significantly improved BSE scores, rising from 48.21 ± 7.40 before discharge to 58.88 ± 5.26 four weeks after discharge^(20)^. Other studies have also reported statistically significant increases in BSES-SF scores between pre- and post-education assessments^(15)^, pre-education and 4 days, 2 weeks, and 6 weeks postpartum^(33)^, as well as pre-education and postpartum day 9 and month 2^(34)^. The BSES-SF scores reported four weeks postpartum in previous studies ranged from 49 to 67^(16,17,19−21,31,32)^. As shown in the findings of this study, breastfeeding education has a positive impact on mothers’ BSE. Healthcare professionals should prioritize regular breastfeeding education in the early postpartum period, particularly for young, primiparous mothers with higher education levels, high incomes, employment, cesarean deliveries, no prior breastfeeding experience, or pregnancy-related health conditions. However, it is recommended that breastfeeding education and follow-ups continue at regular intervals throughout the postpartum period to further support and assess mothers’ breastfeeding success. The strength of this study lies in its longitudinal assessment of the impact of breastfeeding education on BSE. Another notable strength is its examination of the factors influencing mothers’ BSE before discharge and four weeks later, using a larger sample size compared to previous studies. The findings of this study provide valuable insights for healthcare professionals regarding the importance of breastfeeding education programs in helping mothers overcome breastfeeding challenges and determining the optimal timing for education. Additionally, these results may serve as a foundation for future research on diverse populations, contributing to the broader literature on this subject.

This study has some limitations. First, as the sample was drawn from a single city, the findings cannot be generalized to all mothers in Türkiye. Second, since the study was conducted in a baby-friendly hospital, a control group could not be included, and the study was limited to a single-group design. Further research is needed to explore the relationship between BSES-SF and LATCH scores throughout the postpartum period to enhance awareness among healthcare providers and to identify and support mothers at risk of low breastfeeding self-efficacy.

## CONCLUSION

In this study, the mean LATCH score of the mothers was high (9.2 ± 1.3). Additionally, higher LATCH scores were observed in mothers with primary or secondary education, as well as in those who were literate, unemployed, those with three or more pregnancies, and in those with three children. Mothers who breastfed their infants within the first hour and those with previous breastfeeding experience also had higher LATCH scores. Mothers who had no diseases during pregnancy, had prior breastfeeding experience, had four pregnancies, and had three children exhibited higher BSES-SF scores before discharge. Four weeks after discharge, the mean BSES-SF score increased significantly from 58.9 ± 6.7 to 68.4 ± 1.9. A significant increase in BSES-SF scores was observed in mothers with prior breastfeeding experience four weeks after discharge. The breastfeeding education provided to mothers effectively enhanced BSE levels in the postpartum period.
