# Supplementary material for: The factors affecting breastfeeding self-efficacy, LATCH scores and effect of postnatal breastfeeding education on mothers’ self-efficacy
Source: Rev Esc Enferm USP. 2025 May 2;59:e20240281. doi: 10.1590/1980-220X-REEUSP-2024-0281en (PMC12063537; doi:10.1590/1980-220X-REEUSP-2024-0281en)
Supplement: Translation

# Translation: Factores que afectan la autoeficacia en la lactancia materna, puntuaciones LATCH y efecto de la educación posnatal sobre la lactancia materna en la autoeficacia de las madres*

**DOI:** 10.1590/1980-220X-REEUSP-2024-0281es

**Authors:** Aslı Karakuş Selçuk, Emre Yanikkerem, Seda Ünalmiş Erdoğan

**Affiliations:** 1 Manisa Celal Bayar University, Faculty of Health Science, Nursing Department, Manisa, Turkey.; 2 Manisa Celal Bayar University, Hafsa Sultan Hospital, Manisa, Turkey.

**Keywords:** Lactancia Materna, Autoeficacia, Educación, Periodo Posparto

## Abstract

**Objetivo:**

Identificar los factores que afectan la autoeficacia en la lactancia materna y las puntuaciones LATCH y evaluar el impacto de la educación posnatal sobre la lactancia materna en la autoeficacia de las madres.

**Método:**

Este estudio empleó un diseño pretest-postest cuasiexperimental con un solo grupo. Se completó con 217 madres que dieron a luz en un hospital de Turquía entre mayo de 2023 y diciembre de 2023. Los datos se recopilaron utilizando la Escala de autoeficacia en la lactancia materna y la herramienta de evaluación LATCH. Se brindó educación sobre la lactancia materna a las madres antes del alta. La autoeficacia en la lactancia materna se reevaluó por teléfono cuatro semanas después del alta.

**Resultados:**

La puntuación media LATCH entre las madres fue de 9,2 ± 1,3 (mín. = 3, máx. = 10). Se observaron puntuaciones LATCH más altas en las madres con educación primaria o secundaria, las que estaban desempleadas, tenían cuatro o más embarazos e hijos, amamantaron en la primera hora y tenían experiencia previa en lactancia materna antes del alta. Las madres con experiencia en lactancia materna y aquellas con cuatro embarazos y tres hijos tuvieron puntuaciones más altas de autoeficacia en la lactancia materna antes del alta. Cuatro semanas después de que se les dio el alta a las madres, la puntuación media de autoeficacia en la lactancia materna aumentó significativamente de 58,9 ± 6,7 a 68,4 ± 1,9. Se observó una mejora estadísticamente significativa en la autoeficacia en la lactancia materna solo entre las madres con experiencia previa en lactancia materna cuatro semanas después del alta.

**Conclusión:**

La educación regular sobre lactancia materna en el período posparto temprano es crucial, en particular para las madres jóvenes, primíparas con niveles de educación más altos, aquellas que están empleadas, con antecedentes de partos por cesárea o falta de experiencia previa en lactancia materna. Los hallazgos indican que la educación sobre lactancia materna impacta positivamente en la autoeficacia en la lactancia materna de las madres.

## INTRODUCCIÓN

Se reconoce universalmente que la leche materna es la fuente óptima de nutrición para los recién nacidos. El Fondo de las Naciones Unidas para la Infancia (UNICEF) recomienda que las madres inicien la lactancia materna en la primera hora después del nacimiento, amamanten exclusivamente durante los primeros seis meses y continúen amamantando hasta que el niño cumpla al menos dos años o más^(1)^. A pesar de estas ventajas bien documentadas, los datos mundiales indican que solo el 48% de los bebés de 0 a 5 meses son amamantados exclusivamente. Esta tasa varía significativamente según la región, alcanzando el 60% o más en el sur de Asia, pero cayendo al 26% en América delNorte^(2)^. Según los últimos datos de la Encuesta de Salud de la Población de Turquía (2018), aunque la mayoría de los niños menores de dos años (71,0%) fueron amamantados en la primera hora de vida, el 41,0% de los bebés menores de 6 meses fueron amamantados exclusivamente^(3)^.

La autoeficacia se refiere a la percepción que tiene una persona de su capacidad para realizar una acción o comportamiento específico. En el contexto de la lactancia materna, esta percepción se define como autoeficacia en la lactancia materna (BSE, por sus siglas en inglés)^(4)^. La autoeficacia de la madre puede afectar a la iniciación y continuación de la lactancia materna, a cómo afrontará las dificultades en este proceso y a la decisión de la madre de amamantar^(5)^. Estudios nacionales e internacionales han destacado varios factores clave que afectan a la autoeficacia de la lactancia materna en el periodo posparto. Entre ellos se encuentran el número de partos^(6)^, la experiencia previa en la lactancia materna^(7)^, el estado de lactancia materna exclusiva^(7−10)^, la recepción de asesoramiento sobre lactancia materna antes o después del parto^(7)^, el número de hijos^(11)^, el modo de parto (parto vaginal espontáneo)^(12)^, las dificultades de la lactancia materna^(5)^, el patrón de alimentación del recién nacido en las 24 horas anteriores (lactancia exclusiva, parcial o nula)^(5)^, la percepción de insuficiencia de suministro de leche^(5)^ y la actitud del cuidador principal hacia la lactancia materna^(5)^.

La literatura indica que los programas educativos sobre lactancia materna y las intervenciones de apoyo proporcionadas a las madres durante los períodos prenatal^(13,14)^ y posparto^(15−21)^ son eficaces para mejorar las puntuaciones de LATCH y BSE. Los estudios realizados en Turquía han demostrado que la educación sobre lactancia materna impartida mediante el método de enseñanza recíproca^(17)^ y la teleeducación sobre lactancia materna^(16)^ mejoraron el éxito de la lactancia materna y el BSE de las madres. Del mismo modo, una investigación realizada en Japón^(21)^ descubrió que un programa de autocuidado de la lactancia materna aumentaba tanto el BSE como la continuación de la lactancia materna, mientras que estudios realizados en China^(20)^ demostraron que la mejora de la autoeficacia repercutía significativamente en la tasa de lactancia materna exclusiva.

El aspecto clave que diferencia este estudio de investigaciones anteriores es su evaluación tanto de la eficacia de la educación posnatal sobre lactancia materna en la BSE como de los factores que influyen en la BSE, combinada con un gran tamaño de muestra. Los objetivos de este estudio fueron: identificar los factores que afectan a la autoeficacia de la lactancia materna y a las puntuaciones LATCH y evaluar el impacto de la educación posnatal sobre lactancia materna en la autoeficacia de las madres.

### Preguntas de Investigación

- ¿Cómo influyen las características de las madres en las puntuaciones de BSE y LATCH antes del alta?
- ¿Cómo influyen las características de las madres en la BSE cuatro semanas después del alta?
- ¿Existe alguna diferencia en las medidas educativas previas y posteriores a la prueba en las puntuaciones de la BSE?

## MÉTODO

### Diseño del Estudio

Este estudio empleó un diseño cuasi-experimental de prueba previa y posterior con un solo grupo.

### Cálculo del Tamaño de la Muestra

El universo del estudio consistió en 882 madres que dieron a luz en la clínica de obstetricia y ginecología de un hospital universitario de Turquía entre el 1 de mayo de 2023 y el 31 de diciembre de 2023. Se utilizó la fórmula de población universalmente conocida para determinar el tamaño mínimo de la muestra. Utilizando esta fórmula, el tamaño mínimo de la muestra se calculó en 213 mujeres con el software estadístico Epi Info 2000 (intervalo de confianza del 90%, prevalencia desconocida del 50% y margen de error del 5%). El tamaño de la muestra se calculó además de esta manera como 207 (un tamaño de efecto medio de 0,3, una probabilidad de error alfa del 5% y una potencia del 99%) utilizando el programa G*Power. El estudio se completó con 217 madres y se volvió a realizar un análisis de potencia a posteriori. El tamaño del efecto fue de 1,427 y la potencia fue de 1,00^(22)^.

### Criterios de Inclusión y Exclusión

El estudio incluyó a madres mayores de 18 años, que estaban amamantando y que tenían bebés sanos. Se excluyeron del estudio las madres con deficiencias visuales o auditivas, enfermedades psiquiátricas, partos múltiples, bebés que pesaban menos de 2500 g o que nacieron prematuramente, las que no hablaban turco y las que no participaron voluntariamente.

### Herramientas Para Recolectar Datos

El cuestionario constaba de tres secciones. La primera sección incluía 19 preguntas que abarcaban las características sociodemográficas y obstétricas de las madres, como la edad, el nivel de educación, los ingresos, la situación laboral, el modo de parto, el número de partos, el momento de inicio de la lactancia, el peso del recién nacido y el sexo. Este cuestionario se desarrolló a partir de la literatura existente^(6−11)^. La segunda sección incluía la Herramienta de Evaluación de la Lactancia Materna LATCH, una escala desarrollada por Jensen, Wallace y Kelsay^(23)^ para evaluar los comportamientos de lactancia materna de las madres. Cada elemento de la escala consta de cinco criterios, evaluados con puntuaciones de 0, 1 o 2 puntos. LATCH es un acrónimo que representa los componentes clave del diagnóstico y la evaluación de la lactancia materna:

- L (Latch on breast): Cómo se agarra el recién nacido al pecho.
- A (Audible Swallowing): Detectar los sonidos de deglución del recién nacido.
- T (Type of Nipple): Tipo de pezón.
- C (Comfort of Breast/Nipple): La comodidad del pecho/pezón de la madre.
- H (Hold/Position): El nivel de asistencia requerido para que la madre coloque a su recién nacido para que succione^(23,24)^.

La escala LATCH va de 0 a 10, y las puntuaciones más altas indican un mayor éxito de la lactancia materna. La puntuación se determina observando el comportamiento de la madre durante la lactancia. Yenal y Okumuş^(24)^ realizaron un estudio turco de validez y fiabilidad de esta herramienta, con un valor alfa de Cronbach de 0,95, lo que indica una alta fiabilidad. La tercera sección incluía la Escala de autoeficacia para la lactancia materna (BSES-SF, por sus siglas en inglés). Desarrollada originalmente por Dennis y Faux^(25)^ con ٣٣ ítems, la escala se revisó en ٢٠٠٣ y se convirtió en una versión corta de ١٤ ítems^(26)^. Esta escala de tipo Likert de cinco puntos va de ١ (nada seguro) a ٥ (muy seguro), con puntuaciones totales que van de ١٤ a ٧٠. Las puntuaciones más altas indican un mayor BSE. El estudio turco de validez y fiabilidad de la escala fue realizado por Tokat et al.^(4)^, con un valor alfa de Cronbach de 0,86, lo que demuestra una buena fiabilidad.

### Recolección de Datos

Los datos se recolectaron en tres etapas secuenciales, cada una de las cuales tenía un investigador asignado. En la primera etapa, los datos se recolectaron mediante entrevistas cara a cara en las habitaciones de las madres. Durante esta fase de 10 minutos, se registraron los números de teléfono móvil de las madres y se recolectaron datos utilizando el cuestionario de características sociodemográficas y obstétricas y los formularios bses-sf. Se les informó que se pondrían en contacto con ellas cuatro semanas después. En la segunda fase, se evaluó el éxito de la lactancia materna de las mujeres utilizando la escala LATCH, y recibieron educación sobre lactancia materna. En la fase final, se contactó por teléfono a las madres cuatro semanas después del alta y se reevaluó el BSE de las madres con BSES-SF.

### Intervención

En el hospital donde se realizó el estudio, la estancia hospitalaria mínima es de 24 horas para las madres que dan a luz por vía vaginal y de 48 horas para las que se someten a una cesárea. En este hospital amigo del bebé, las madres comienzan a amamantar a sus recién nacidos en los 30 minutos posteriores al nacimiento, todas las madres reciben educación sobre lactancia y hay apoyo disponible durante toda su estancia en el hospital. Se siguió el mismo programa estructurado de educación sobre lactancia, desarrollado a partir de la literatura^(1,2,20,27)^, para cada madre. El tercer investigador impartió una conferencia de 20 minutos, que incluía información sobre la estructura de los senos, el mecanismo de lactancia, la posición de agarre, la producción de leche, las técnicas de relajación para los bebés, la importancia y composición de la leche materna, los beneficios de la lactancia materna tanto para la madre como para el bebé, la frecuencia y duración de la lactancia materna, los problemas comunes de la lactancia materna y las soluciones sugeridas, las necesidades nutricionales de las madres lactantes y la extracción y almacenamiento de la leche materna. Los temas tratados se basaron en las preguntas más comunes planteadas por las madres durante el período posparto temprano. A través de la educación, se dirigió la atención de las madres a sus conocimientos y habilidades que facilitan una lactancia exitosa (persuasión verbal) y cómo podrían lograr experiencias exitosas de lactancia (logros de rendimiento) aplicando estas habilidades. Se demostraron las técnicas de lactancia materna de forma práctica y se animó a las madres a hacer preguntas. Finalmente, el investigador proporcionó comentarios positivos (persuasión verbal) utilizando sus conocimientos y experiencia profesionales. La tercera investigadora, una enfermera especializada en lactancia que trabaja en esta clínica, recibe formación periódica sobre lactancia. Visitó todas las habitaciones de las madres durante su estancia en el hospital, proporcionando educación individualizada sobre lactancia el primer día después del parto.

### Análisis de datos y Tratamiento

El análisis estadístico se realizó utilizando el programa SPSS 20,0. Se utilizaron distribuciones de frecuencias para evaluar las características descriptivas. La distribución normal de los datos se determinó mediante pruebas de asimetría y curtosis. Dado que los datos no seguían una distribución normal, la relación entre las variables independientes del estudio y las puntuaciones de LATCH y BSE se evaluó con las pruebas de Kruskal Wallis, U de Mann-Whitney y T2 de Tamhane. La prueba de Wilcoxon se utilizó para determinar la diferencia en las medidas previas y posteriores a la prueba en las puntuaciones de BSE. Un nivel p de < 0,05 se consideró estadísticamente significativo.

### Aspectos Éticos

Se obtuvo la aprobación ética del Comité Ético de la Universidad Celal Bayar de Manisa (Fecha: 12,04,2023, Número: 20,478,486/1803). Se obtuvo permiso de la institución para recolectar los datos para el estudio. Se obtuvo el consentimiento informado escrito y verbal de todas las madres. Se recibió permiso de Yenal para la versión turca de BSES-SF y de Tokat para la versión turca de LATCH para usar en este estudio.

## RESULTADOS

En el estudio, se excluyeron 648 de las 882 madres que dieron a luz en el hospital durante el período de recolección de datos. En la primera etapa, se recolectaron datos de 234 madres, y en la segunda etapa, se proporcionó educación sobre lactancia. En la tercera etapa, se excluyó del estudio a las madres que no respondieron a las llamadas telefónicas (n = 9) y a las que no amamantaron (n = 8). El estudio se completó con 217 madres. En la Figura 1 se presenta un diagrama que muestra el flujo de participantes.

**Figura 1:** Diagrama de flujo de los participantes en el estudio.

### Características Descriptivas de las Madres

En el estudio, la edad media de las madres era de 28,8 ± 5,5 (mín = 18, máx = 43), y aproximadamente la mitad de las madres (47,0%) tenían entre 26 y 33 años. En general, el 43,8% tenía estudios primarios o secundarios, el 79,3% estaba en paro y el 68,2% declaró que sus ingresos familiares eran iguales a sus gastos. La mayoría de las madres (86,2%) tuvieron embarazos planificados, el 88,0% tuvieron un parto por cesárea, cerca de la mitad de las madres experimentaban su primer embarazo (41,9%), eran primíparas (47,5%) y el 59,4% tuvieron enfermedades durante el embarazo. El peso medio de los recién nacidos al nacer fue de 3175,9 ± 480,9 (mín = 2500, máx = 4600). El 86,2% de las madres amamantaron a sus hijos durante la primera hora. La mayoría de las madres (81,1%) declaró no haber recibido formación sobre lactancia materna, y el 46,1% tenía experiencia previa en este campo (Tabla 1).

**Tabla 1:** Características descriptivas de las madres – Manisa, Turquía, 2023.

Características de las madres	n	%
**Grupos de edad de las madres**		
18–25 años	66	30,4
26–33 años	102	47,0
34–43 años	49	22,6
**Nivel educativo de las madres**		
Alfabetizado/No	9	4,1
Escuela primaria/secundaria	95	43,8
Bachillerato	61	28,1
Universitarios y superiores	52	24,0
**Situación laboral de las madres**		
Empleado	45	20,7
Desempleado	172	79,3
**Ingresos**		
Los ingresos son inferiores a los gastos	54	24,9
Los ingresos son iguales a los gastos	148	68,2
Los ingresos son superiores a los gastos	15	6,9
**Número de embarazos**		
1	91	41,9
2	41	18,9
3	40	18,4
4 o más	45	20,7
**Número de niños**		
1	103	47,5
2	44	20,3
3	41	18,9
4 o más	29	13,4
**Tipo de parto**		
Parto por cesárea	191	88,0
Parto vaginal	26	12,0
**Sexo del recién nacido**		
Mujer	102	47,0
Hombre	115	53,0
**Peso del recién nacido al nacer**		
2500–3000	82	37,8
3001–3500	93	42,9
3501 o más	42	19,4
**Embarazo actual**		
Planificado	187	86,2
No planificado	30	13,8
**Tuvo una enfermedad durante el embarazo**		
Sí	88	40,6
No	129	59,4
**Recibió algún tipo de formación sobre lactancia materna**		
Sí	41	18,9
No	176	81,1
**Experiencias de lactancia materna de las madres**		
Sí	100	46,1
No	117	53,9
**Momento de la lactancia materna**		
Primera hora	187	86,2
Más de 1 hora	30	13,8
**Total**	**217**	**100,0**

### Relación Entre las Características Descriptivas de las Madres y la Puntuación Latch Antes del Alta

En este estudio, la puntuación LATCH media de las madres fue de 9,2 ± 1,3 (mín = 3, máx = 10). La puntuación media de LATCH fue significativamente mayor en las madres con estudios primarios o secundarios y en las alfabetizadas o analfabetas, en comparación con las que tenían estudios universitarios o superiores (p = 0,002). Las madres desempleadas tenían puntuaciones LATCH más altas que las empleadas (p = 0,001). La puntuación LATCH fue significativamente mayor en las madres que tuvieron tres o cuatro o más embarazos en comparación con las que tuvieron uno o dos embarazos (p = 0,000). Del mismo modo, la puntuación media del LATCH fue mayor en las madres que tuvieron tres hijos que en las que tuvieron uno o dos hijos (p = 0,000). Las madres que amamantaron a sus hijos durante la primera hora (p = 0,044) y las que tenían experiencia previa en lactancia materna (p = 0,000) obtuvieron puntuaciones LATCH más altas (Tabla 2).

**Tabla 2:** Relación entre las características descriptivas de las madres y las puntuaciones LATCH antes del alta – Manisa, Turquía, 2023.

Características de las madres (n: 217)	Puntuaciones LATCH antes del alta				
	Media	DE	Mediana	IIC	Prueba/p
**Grupos de edad de las madres**					
18–25 años	9,02	1,26	9,00	1,00	KW = 3,444
26–33 años	9,19	1,27	10,00	1,00	p = 0,179
34–43 años	9,31	1,21	10,00	1,00
**Nivel educativo de las madres**					
Alfabetizado/No analfabeto (a)	9,67	0,50	10,00	1,00	KW = 14,921
Escuela primaria/secundaria (b)	9,43	1,02	10,00	1,00	**p = 0,002**
Bachillerato (c)	9,13	1,19	10,00	1,00	*a > d, b > d
Universidad y superiores (d)	8,63	1,60	9,00	2,00	
**Ingresos**					
Los ingresos son inferiores a los gastos	9,31	1,11	10,00	1,00	KW = 1,778
Los ingresos son iguales a los gastos	9,14	1,30	10,00	1,00	p = 0,411
Los ingresos son superiores a los gastos	8,93	1,22	9,00	2,00	
**Situación laboral de las madres**					
Empleado	8,60	1,49	9,00	2,00	MU = 2693,000
Desempleado	9,31	1,14	10,00	1,00	**p = 0,001**
**Embarazo actual**					
Planificado	9,12	1,29	10,00	1,00	MU = 2357,000
No planificado	9,47	0,97	10,00	1,00	p = 0,121
**El tipo de parto**					
Parto por cesárea	9,15	1,26	10,00	1,00	MU = 2275,500
Parto vaginal	9,30	1,19	10,00	1,00	p = 0,445
**El número de embarazos**					
1 (a)	8,66	1,49	9,00	2,00	KW = 43,512
2 (b)	9,12	1,08	9,00	1,00	**p = 0,000**
3 (c)	9,75	0,59	10,00	0,00	*c > a, c > b, d > a, d > b
4 o más (d)	9,71	0,79	10,00	0,00	
**El número de niños**					
1 (a)	8,72	1,47	9,00	2,00	KW = 36,243
2 (b)	9,27	1,02	10,00	1,00	**p = 0,000**
3 (c)	9,78	0,57	10,00	0,00	*c > a, c > b, d > a
4 o más (d)	9,69	0,81	10,00	0,00	
**Tuvo una enfermedad durante el embarazo**					
Sí	9,05	1,28	9,50	1,00	MU = 5114,500
No	9,25	1,24	10,00	1,00	p = 0,172
**Sexo del recién nacido**					
Mujer	9,18	1,30	10,00	1,00	MU = 5703,500
Hombre	9,16	1,22	10,00	1,00	p = 0,699
**Peso del recién nacido al nacer**					
2500–3000	9,09	1,28	9,00	1,00	KW = 1,872
3001–3500	9,17	1,32	10,00	1,00	p = 0,392
3501 o más	9,31	1,07	10,00	1,00	
**Momento de la lactancia materna**					
Primera hora	9,23	1,20	10,00	1,00	MU = 2222,000
Más de 1 hora	8,77	1,50	9,00	1,25	**p = 0,044**
**Recibió algún tipo de formación sobre lactancia materna**					
Sí	9,02	1,37	9,00	1,00	MU = 3285,000
No	9,19	1,23	10,00	1,00	p = 0,324
**Experiencias de lactancia materna de las madres**					
Sí	9,63	0,73	10,00	1,00	MU = 3570,000
No	8,77	1,46	9,00	2,00	**p = 0,000**

IIC: Intervalo Intercuartílico, KW: Kruskal Wallis (test para muestras independientes), MU: U de Mann-Whitney (prueba de dos muestras independientes)

*Tamhane’s T2, DE: Desviación estándar.

### Relación Entre las Características Descriptivas de las Madres y las Puntuaciones Bses-Sf Antes y Cuatro Semanas Después del Alta

Se observó que las madres que no habían padecido enfermedades durante el embarazo (p = 0,009), y las que tenían experiencia previa en lactancia materna (p = 0,005), cuatro embarazos (p = 0,011) y tres hijos (p = 0,007) tenían puntuaciones BSES-SF más altas antes del alta. No hubo diferencias significativas entre las puntuaciones BSES-SF y los grupos de edad, el nivel educativo, los ingresos percibidos, la situación laboral, el tipo de parto, el sexo del recién nacido, el peso al nacer o el momento de inicio de la lactancia materna antes del alta. Sólo se encontró una diferencia significativa en las puntuaciones BSES-SF en las madres con experiencia previa en lactancia materna (p = 0,003) cuatro semanas después del alta (Tabla 3).

**Tabla 3:** Relación entre las características descriptivas de las madres y las puntuaciones BSES-SF antes y cuatro semanas después del alta – Manisa, Turquía, 2023.

Características de las madres (n: 217)	Puntuación BSES-SF										
	Antes del alta					Cuatro semanas después del alta				
	Media	DE	Mediana	IIC	Prueba/p	Media	DE	Mediana	IIC	Prueba/p
**Grupos de edad de las madres**										
18–25 años	58,08	6,38	56,00	10,25	KW = 1,658	68,36	1,76	68,00	2,00	KW = 0,757
26–33 años	59,06	6,82	56,00	11,25	p = 0,436	68,54	1,79	68,00	2,00	p = 0,685
34–43 años	59,63	6,99	57,00	11,50		68,08	232	68,00	3,00	
**Nivel educativo de las madres**										
Alfabetizado/No	60,44	7,23	58,00	14,00	KW = 3,003	67,67	4,12	68,00	2,00	KW = 3,358
Primaria/Secundaria	59,22	6,85	56,00	13,00	p = 0,391	68,64	1,62	70,00	2,00	P = 0340
Bachillerato	59,09	6,60	56,00	11,00		68,28	2,02	68,00	2,50	
Universitarios y superiores	57,77	6,57	56,00	10,75		68,15	1,71	68,00	2,00	
**Ingresos**										
Los ingresos son inferiores a los gastos	59,39	7,01	59,00	12,25	KW = 0,452	68,06	2,32	68,00	3,00	KW = 2,549
Los ingresos son iguales a los gastos	58,82	6,58	56,00	11,00	p = 0,798	68,44	1,79	68,00	2,00	p = 0,221
Los ingresos son superiores a los gastos	57,73	7,43	56,00	13,00		69,00	1,25	70,00	2,00	
**Situación laboral de las madres**										
Empleado	57,27	6,33	56,00	10,00	MU = 3220,000	68,09	2,03	68,00	2,00	MU = 3497,500
Desempleado	59,31	6,78	56,00	11,75	p = 0,082	68,46	1,88	68,00	2,00	p = 0,289
**Embarazo actual**										
Planificado	58,66	6,59	56,00	11,00	MU = 2415,000	68,37	1,86	68,00	2,00	MU = 2593,500
No planificado	60,30	7,46	57,50	13,75	p = 0,221	68,43	2,27	70,00	2,25	p = 0,480
**Tipo de parto**										
Parto por cesárea	58,62	6,71	56,00	11,00	MU = 2037,000	68,36	1,95	68,00	2,00	MU = 2417,000
Parto vaginal	60,85	6,58	62,00	13,25	p = 0,136	68,54	1,68	69,00	3,00	p = 0,815
**Número de embarazos**										
1 (a)	57,49	6,30	56,00	10,00	KW = 11,046	68,25	1,65	68,00	2,00	KW = 5,407
2 (b)	58,61	7,09	56,00	13,00	**p = 0,011**	68,15	238	68,00	2,00	p = 0,144
3 (c)	60,20	6,91	58,00	12,00	*d > a	68,70	2,11	70,00	2,00	
4 o más (d)	60,80	6,58	59,00	13,00		68,58	1,75	70,00	2,00	
**Número de niños**										
1 (a)	57,75	6,37	56,00	11,00	KW = 12,182	68,16	2,02	68,00	2,00	KW = 3,632
2 (b)	58,27	6,76	56,00	12,50	**p = 0,007**	68,70	1,44	68,00	2,00	p = 0304
3 (c)	6131	6,95	61,00	14,00	*c > a	68,46	2,15	70,00	2,50	
4 o más (d)	60,45	6,71	57,00	13,00		68,59	1,78	70,00	2,00	
**Tuvo una enfermedad durante el embarazo**										
Sí	57,49	6,74	55,50	10,75	MU = 4499,000	68,24	1,75	68,00	2,00	MU = 5029,000
No	59,85	6,57	57,00	12,50	**p = 0,009**	68,48	2,01	69,00	2,00	p = 0,128
**Sexo del recién nacido**										
Mujer	58,82	6,46	56,00	11,00	MU = 5740,000	68,56	1,85	68,00	2,00	MU = 5324,500
Hombre	58,95	6,97	56,00	12,00	p = 0,786	68,23	1,96	68,00	2,00	p = 0,212
**Peso del recién nacido al nacer**										
2500–3000	59,49	6,95	57,00	12,00	KW = 1,436	68,33	2,18	68,00	2,00	KW = 0,056
3001–3500	58,43	6,49	56,00	11,50	p = 0,488	68,43	1,75	68,00	2,00	p = 0,973
3501 o más	58,74	6,84	56,00	11,25		68,38	1,74	68,00	2,00	
**Momento de la lactancia materna**										
Primera hora	58,89	6,78	56,00	11,00	MU = 2762,500	68,41	1,87	68,00	2,00	MU = 2663,500
Más de 1 hora	58,87	6,44	56,00	11,00	p = 0,894	68,17	2,21	68,00	2,25	p = 0,636
**Recibió algún tipo de formación sobre lactancia materna**										
Sí	59,05	6,93	57,00	12,00	MU = 3606,500	68,34	2,42	69,00	2,00	MU = 3439,000
No	58,85	6,69	56,00	11,00	p = 0,997	68,39	1,78	68,00	2,00	p = 0,618
**Experiencias de lactancia materna de las madres**										
Sí	60,17	6,95	56,50	12,75	MU = 4545,000	68,74	1,74	70,00	2,00	MU = 4585,000
No	57,79	6,35	56,00	11,00	**p = 0,005**	68,08	2,00	68,00	2,00	**p = 0,003**

IIC: Intervalo Intercuartílico, KW: Kruskal Wallis (test para muestras independientes), MU: Mann-Whitney U (test para dos muestras independientes)

*Tamhane’s T2, DE: Desvío estándar.

### Puntuaciones Bses-Sf de las Madres Antes y Cuatro Semanas Después del Alta

En este estudio, mientras que la puntuación BSES-SF de las madres era de 58,9 ± 6,7 (mín = 45, máx = 70) antes del entrenamiento en lactancia materna, aumentó de forma estadísticamente significativa a 68,4 ± 1,9 (mín = 57, máx = 70) cuatro semanas después del alta (Datos no mostrados). Hubo una diferencia significativa entre cuatro semanas después del alta y antes del alta en las puntuaciones de EEB de las madres tras realizar la prueba de Wilcoxon (Z = −11,936 p = 0,000) y los resultados de la EEB de las madres también indican una ganancia positiva (Tabla 4).

**Tabla 4:** Puntuaciones BSES-SF de las madres antes y cuatro semanas después del alta – Manisa, Turquía, 2023.

BSE cuatro semanas después del alta-BSE antes del alta (n: 217)	n	Rango medio	Suma de rangos	
Rangos negativos	10	19,50	195,00	
Rangos positivos	187	103,25	19308,00	Z = -11.
Lazos	20			* **p = 0,000**

*Prueba de Wilcoxon.

## DISCUSIÓN

El presente estudio tenía como objetivo identificar los factores que influyen en las puntuaciones de BSE y LATCH y examinar el impacto de la educación postnatal sobre lactancia materna en la autoeficacia de las madres. En este estudio, la puntuación media de LATCH fue de 9,2 ± 1,3 (mín = 3, máx = 10). Teniendo en cuenta que la escala LATCH va de 0 a 10, los resultados indican que las puntuaciones medias LATCH de las madres fueron altas. Una revisión de los estudios realizados en Turquía reveló que las puntuaciones medias de las madres en la escala LATCH eran de 6,2 ± 1,6^(7)^, 7,3 ± 2,2^(12)^, 8,2 ± 1,8^(6)^ y 8,4 ± 1,5^(11)^. De forma similar, estudios de la India e Italia informaron de puntuaciones medias de LATCH de 7,2 ± 1,1^(28)^ y 7,3 ± 1,7^(29)^, respectivamente. En comparación con estos estudios, las puntuaciones LATCH en el presente estudio fueron más altas, lo que puede atribuirse al hecho de que el estudio se realizó en un hospital amigo de los niños. Se cree que la educación sobre lactancia materna proporcionada a las madres en la clínica contribuyó a este aumento de las puntuaciones LATCH. Además, la enfermera del departamento de lactancia materna (la tercera investigadora) que trabaja en esta clínica recibe regularmente formación sobre lactancia materna y tiene amplia experiencia en proporcionar educación práctica sobre lactancia materna y apoyo a las madres. En consonancia con nuestros hallazgos, estudios anteriores han demostrado que las madres con cuatro o más embarazos^(6)^, las madres con tres hijos^(7,11)^, las madres con experiencia previa en lactancia materna antes del alta^(7)^ y las madres que amamantaban exclusivamente antes del alta^(28,29)^ tenían puntuaciones LATCH más altas. Estos resultados ponen de relieve el papel crucial de la experiencia materna en el éxito de la lactancia. La experiencia previa en lactancia materna aumenta la confianza en sí misma, y la familiaridad con las técnicas de lactancia materna conduce a mejores resultados en la lactancia materna. En el presente estudio, las que iniciaron la lactancia en la primera hora obtuvieron puntuaciones LATCH más altas que las que la iniciaron más tarde. Del mismo modo, Gercek et al.^(6)^ informaron de que las que iniciaron la lactancia materna en los 30 minutos posteriores al nacimiento tenían puntuaciones LATCH más altas que las que la iniciaron entre 1 y 4 horas o 5 o más horas después del nacimiento. Este inicio temprano de la lactancia materna puede contribuir a un vínculo materno-infantil más fuerte, un entorno de lactancia materna más propicio y una mayor motivación materna para una lactancia materna exitosa. Además, las madres con estudios primarios o secundarios, las alfabetizadas o no analfabetas y las desempleadas obtuvieron puntuaciones LATCH significativamente más altas desde el punto de vista estadístico, debido a que las mujeres de estos grupos tenían actitudes más positivas hacia la lactancia materna y a que las madres desempleadas disponen de más tiempo y oportunidades para amamantar a sus hijos. Como se ha observado en estudios anteriores^(6,7,11)^, es un resultado esperado que las madres con embarazos anteriores, hijos y experiencia en lactancia materna demuestren un mayor éxito en la lactancia materna y puntuaciones LATCH más altas.

En el presente estudio, antes de la educación proporcionada, la puntuación media BSES-SF de las madres fue de 58,9 ± 6,7 (mín = 45, máx = 70). En algunos estudios realizados en Turquía, la puntuación BSES-SF oscilaba entre 54 y 57^(6,7,11,15,30)^. La puntuación media del BSES-SF fue de 59,7 ± 6,3 en Brasil^(19)^, 48,2 ± 7,4 en China^(20)^, 47,3 ± 8,6 en Canadá^(31)^ y 34,8 ± 1,1 en Japón^(21)^. Los resultados sugieren que la puntuación BSES-SF de este estudio coincide con la de estudios anteriores realizados en Turquía. Sin embargo, las puntuaciones BSES-SF más bajas registradas en estudios de otros países pueden atribuirse a diferencias culturales y sociodemográficas. En consonancia con nuestro estudio, las madres con cuatro o más embarazos^(6)^ y las que tenían tres o más hijos^(7,11)^ presentaban puntuaciones BSES-SF más altas antes del alta. Además, se observó un aumento significativo de las puntuaciones del BSE en las madres con experiencia previa en lactancia materna cuatro semanas después del alta. El BSE está influido por la experiencia materna y los antecedentes culturales, y tiende a ser mayor en las mujeres con embarazos múltiples. Las diferencias en las tasas de lactancia materna y autoeficacia entre países pueden estar relacionadas con variaciones en las estructuras sociales y los sistemas de apoyo sanitario.

Los resultados de este estudio demostraron un aumento significativo en la puntuación media del BSES-SF cuatro semanas después del alta (antes del alta: 58,9 ± 6,7, cuatro semanas después del alta: 68,4 ± 1,9). En la bibliografía, se realizaron estudios con pretest-postest aleatorizados controlados cuasiexperimentales en diferentes intervalos de tiempo de seguimiento para evaluar el BSE de las madres. Estudios previos aleatorizados controlados y cuasiexperimentales con diseños pretest-postest realizados en Canadá, China, Brasil, Japón, Egipto y Turquía también han encontrado que la educación sobre lactancia materna mejora significativamente los niveles de BSE durante el periodo postparto^(16,17,19-21,31-34)^. Los estudios realizados en Turquía muestran que tras la educación sobre lactancia materna mediante el método teach-back, la puntuación media del BSES-SF aumentó de 45,67 ± 11,65 antes de la educación a 67,02 ± 2,16^(17)^. Del mismo modo, tras la teleeducación sobre lactancia materna, la puntuación BSES-SF aumentó de 43,54 ± 7,15 a 52,04 ± 6,25 cuatro semanas después^(16)^. Un estudio cuasiexperimental pretest-postest en Japón^(21)^ también descubrió que un programa de autocuidado de la lactancia, que incluía un folleto y materiales audiovisuales, aumentaba eficazmente las puntuaciones del BSE de 34,8 ± 1,1 antes de la educación a 49,9 ± 0,9 cuatro semanas después. En China, un seguimiento telefónico postnatal mejoró significativamente las puntuaciones de BSE, pasando de 48,21 ± 7,40 antes del alta a 58,88 ± 5,26 cuatro semanas después del alta^(20)^. Otros estudios también han informado de aumentos estadísticamente significativos en las puntuaciones del BSES-SF entre las evaluaciones previas y posteriores a la educación^(15)^, la educación previa y 4 días, 2 semanas y 6 semanas después del parto^(33)^, así como la educación previa y el día 9 y el mes 2 después del parto^(34)^. Las puntuaciones del BSES-SF comunicadas cuatro semanas después del parto en estudios anteriores oscilaban entre 49 y 67^(16,17,19−21,31,32)^.

Como muestran los resultados de este estudio, la educación sobre la lactancia materna tiene un impacto positivo en el BSE de las madres. Los profesionales sanitarios deberían dar prioridad a la educación regular sobre lactancia materna en el periodo posparto temprano, especialmente en el caso de madres jóvenes primíparas con niveles educativos más altos, ingresos altos, empleo, partos por cesárea, sin experiencia previa en lactancia materna o con problemas de salud relacionados con el embarazo. Sin embargo, se recomienda que la educación y el seguimiento de la lactancia materna continúen a intervalos regulares durante el periodo posparto para seguir apoyando y evaluando el éxito de la lactancia materna de las madres. La fuerza de este estudio reside en su evaluación longitudinal del impacto de la educación en lactancia materna sobre la BSE. Otro punto fuerte notable es su examen de los factores que influyen en la BSE de las madres antes del alta y cuatro semanas después, utilizando una muestra de mayor tamaño en comparación con estudios anteriores. Los resultados de este estudio proporcionan información valiosa para los profesionales sanitarios sobre la importancia de los programas de educación sobre lactancia materna para ayudar a las madres a superar los retos de la lactancia materna y determinar el momento óptimo para la educación. Además, estos resultados pueden servir como base para futuras investigaciones en diversas poblaciones, contribuyendo a la literatura más amplia sobre este tema.

Este estudio tiene algunas limitaciones. En primer lugar, como la muestra procedía de una sola ciudad, los resultados no pueden generalizarse a todas las madres de Turquía. En segundo lugar, como el estudio se llevó a cabo en un hospital amigo de los niños, no se pudo incluir un grupo de control, y el estudio se limitó a un diseño de grupo único. Se necesita más investigación para explorar la relación entre las puntuaciones BSES-SF y LATCH a lo largo del periodo posparto para aumentar la concienciación entre los profesionales sanitarios e identificar y apoyar a las madres en riesgo de baja autoeficacia en la lactancia.

## CONCLUSIÓN

En este estudio, la puntuación media de LATCH de las madres fue alta (9,2 ± 1,3). Además, se observaron puntuaciones LATCH más altas en las madres con estudios primarios o secundarios, así como en las alfabetizadas, desempleadas, con tres o más embarazos y con tres hijos. Las madres que amamantaron a sus hijos durante la primera hora y las que tenían experiencia previa en lactancia materna también obtuvieron puntuaciones más altas en el LATCH. Las madres que no habían padecido enfermedades durante el embarazo, las que tenían experiencia previa en lactancia materna, las que habían tenido cuatro embarazos y las que tenían tres hijos presentaban puntuaciones BSES-SF más altas antes del alta. Cuatro semanas después del alta, la puntuación media del BSES-SF aumentó significativamente de 58,9 ± 6,7 a 68,4 ± 1,9 puntos. Se observó un aumento significativo de las puntuaciones BSES-SF en las madres con experiencia previa en lactancia materna cuatro semanas después del alta. La educación sobre lactancia materna proporcionada a las madres mejoró eficazmente los niveles de BSE en el periodo posparto.
